# Supplementary material for: Genetic Authentication of the Medicinal Plant Portulaca oleracea Using a Quick, Precise, and Sensitive Isothermal DNA Amplification Assay
Source: Int J Mol Sci. 2023 Jun 27;24(13):10730. doi: 10.3390/ijms241310730 (PMC10341654; doi:10.3390/ijms241310730)
Supplement: Supplementary file 1 [file ijms-24-10730-s001.zip › ijms-2431649-supplementary.pdf]

International Journal of
Molecular Sciences

MDPI

Primer set	Primer name	Primer sequence(5'-3')	Length	Tm	GC%
PO01	F3	GCGCGGCTGGCCTAAAAT	18	52.6	61
	B3	CTTAAACTCAGCGGGTAGCC	20	53.8	55
	FIP	TAAACAGGCCGTTAAGCCTCGTTTTTACGACGAC-GAGCTGTTGT	44	69.2	48
	BIP	CTGGAGCACGCTGTTGGGATTTTTTAACGGTTTTGCGTGGCTT	43	69.3	49
PO03	F3	TTACATCGCGCCGCTGGA	18	52.6	61
	B3	CCCGATTTTAAAGCTGGGCT	20	51.8	50
	FIP	GGTCGCAACGGTTTTGCGTGTTTTTTGTTGGGATGGGCTTGTTG	44	70.1	50
	BIP	GGCTACCCGCTGAGTTTAAGCATTTTTTCGCCGTTACTAGGG-GAATC	47	70.7	49

Table S1. The primer used in LAMP assay for *P. oleracea* authentication.

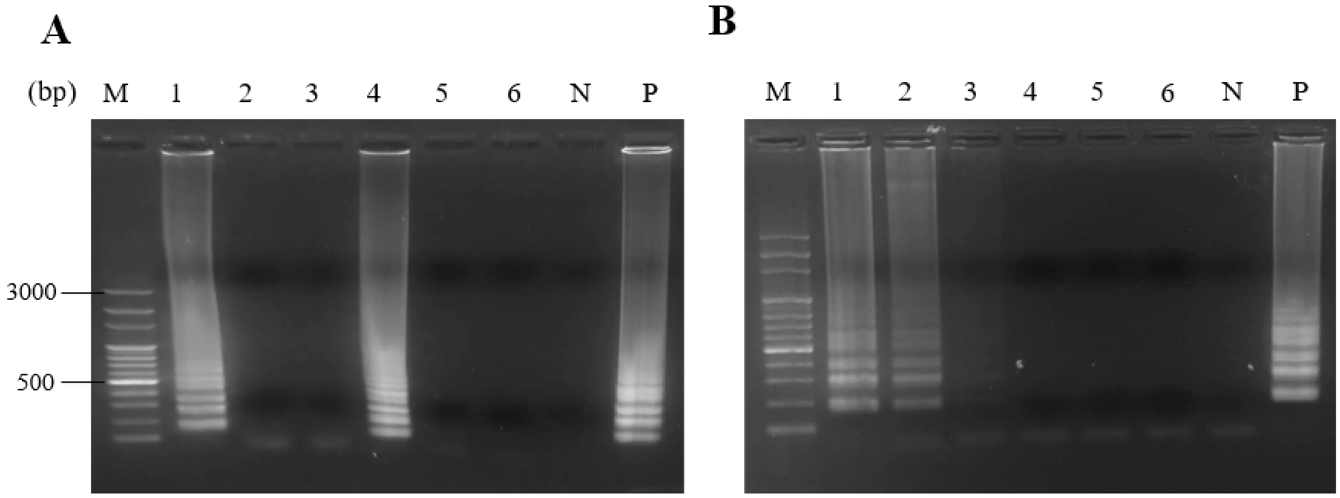

Figure S1. The specificity of *P. oleracea* authentication using the LAMP assay. The specificity of the LAMP reaction was examined using the LAMP primers PO01 (A) and PO03 (B). The annealing position for the PO01 and PO03 LAMP primer set is respectively depicted in the **Fig. S4** and **Fig. S5**. Lane M, DNA marker; Lanes 1-6 indicate *P. oleracea* (PO), *P. umbraticola* (PU), *P. psammotropha* (PPS), *P. pilosa* (PPI), *P. quadrifida* (PQ), and *B. monnieri* (BM); Lane N, negative control; Lane P, positive control.

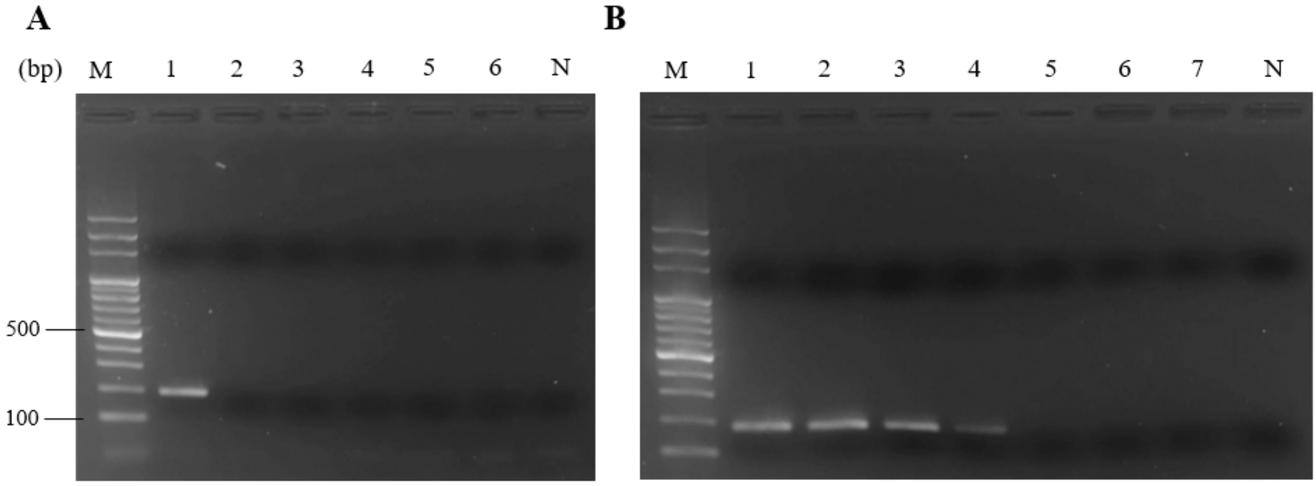

Figure S2. Specificity (A) and sensitivity (B) analysis of species-specific PCR for *Portulaca oleracea* authentication. PCR assays were performed as described in the Materials and Methods section. The PO plant and its related adulterants were used for the determination of specificity by PCR assay (**A**). Lane M, 100 bp DNA ladder marker; Lanes 1-6 indicate the PO, PU, PPS, PPI, PQ and BM plants, respectively; Lane N, negative control. (B) Analysis of the sensitivity of PCR for PO authentication using species-specific PCR primers. Lane M, 100 bp DNA ladder marker; Lanes 1-7 represent 10 ng, 1 ng, 100 pg, 10 pg, 1 pg, 100 fg and 10 fg of DNA used as templates for the PCR assay; Lane N, negative control.

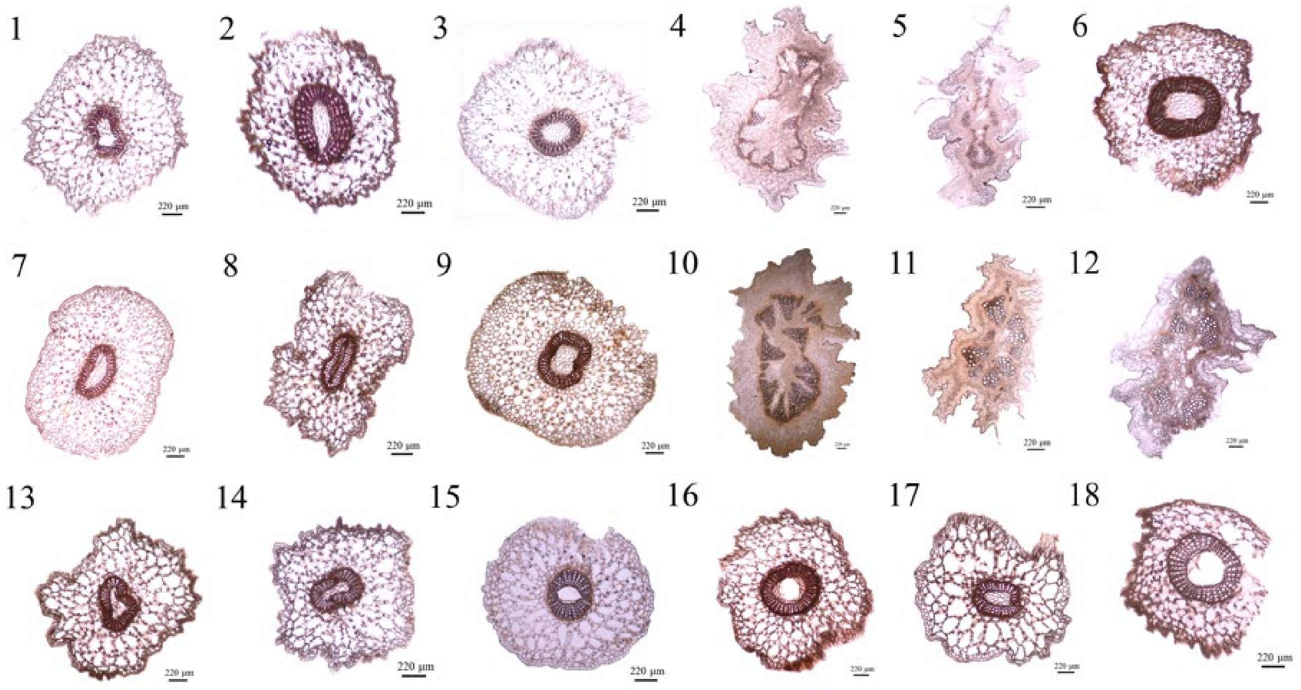

Figure S3. Microscopy identification of transverse sections of commercial PO samples collected from various herbal markets. Eighteen dried commercial PO samples from No. 1 to 18, as described in Fig. 7, were used to produce transverse sections for microscopic examination for PO authentication.

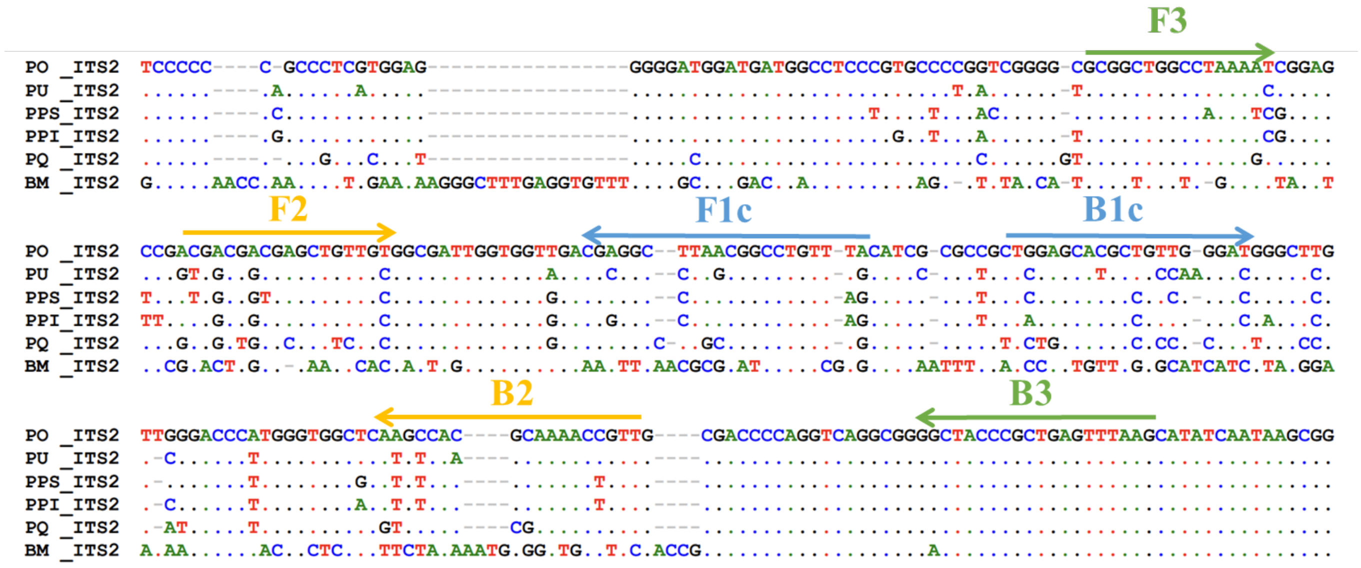

Figure S4. The annealing position for the designed LAMP primer set is depicted in the alignment of the consensus sequence of ITS2. The arrow symbols indicate the direction of DNA polymerization from the PO01 LAMP primer set, which includes outer primers F3 and B3, inner primers FIP (F2+F1c), and BIP (B2+B1c). Differently colored arrows indicate annealing sites on the different strands. The hyphen symbols indicate deleted nucleotides in the sequences. Dots with different colors indicate the same nucleotide between sequences.

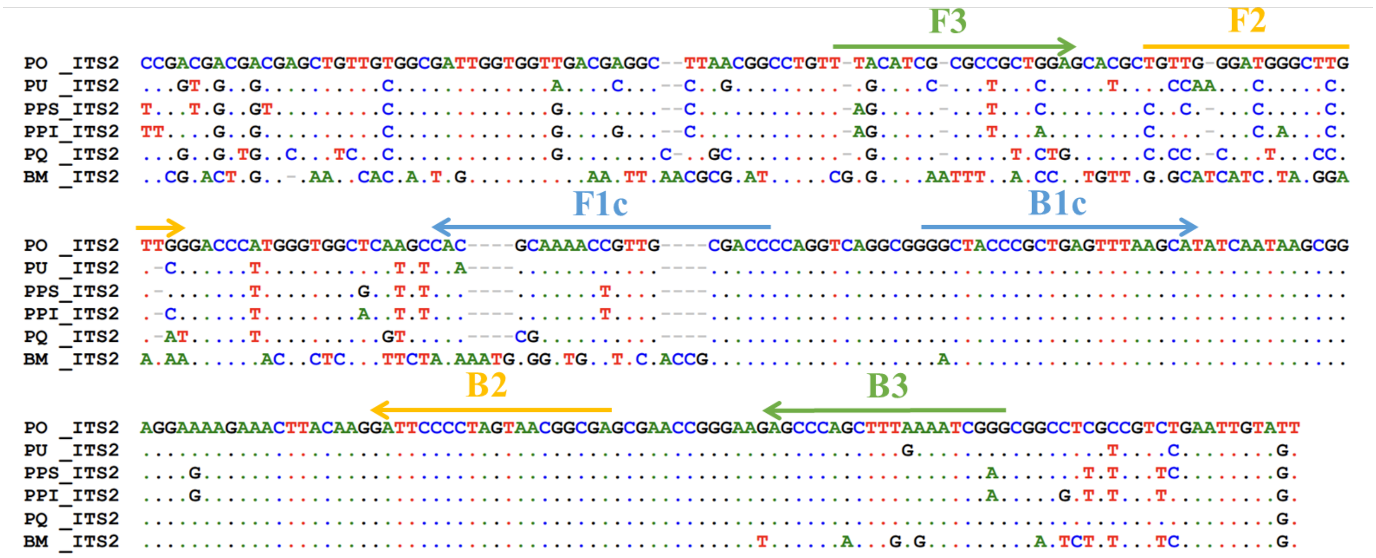

Figure S5. The annealing position for the designed LAMP primer set is depicted in the alignment of the consensus sequence of ITS2. The arrow symbols indicate the direction of DNA polymerization from the PO03 LAMP primer set, which includes outer primers F3 and B3, inner primers FIP (F2+F1c), and BIP (B2+B1c). Differently colored arrows indicate annealing sites on the different strands. The hyphen symbols indicate deleted nucleotides in the sequences. Dots with different colors indicate the same nucleotide between sequences.
